# Relationship Between Thyroid Dysfunction and Ovarian Cancer

**DOI:** 10.3390/biom15060870

**Published:** 2025-06-14

**Authors:** Justyna Gogola-Mruk, Aleksandra Sirek, Izabela Kumor, Gabriela Wojtaszek, Klaudia Roszak, Karolina Kulig, Anna Ptak

**Affiliations:** Laboratory of Physiology and Toxicology of Reproduction, Institute of Zoology and Biomedical Research, Faculty of Biology, Jagiellonian University, Gronostajowa 9, PL 30387 Cracow, Poland; aleksandra.sirek@student.uj.edu.pl (A.S.); izabela.kumor@student.uj.edu.pl (I.K.); gabriela.wojtaszek@student.uj.edu.pl (G.W.); klaudia.roszak@student.uj.edu.pl (K.R.); karolina.m.kulig@student.uj.edu.pl (K.K.)

**Keywords:** hypothyroidism, hyperthyroidism, ovarian cancer, thyroid hormones, thyroid hormone receptors

## Abstract

This review looks at the causes of the association between thyroid dysfunction (hyperthyroidism and hypothyroidism) and ovarian cancer (OC) risk. Epidemiological data have revealed that thyroid dysfunction, particularly hyperthyroidism, is associated with increased risk, progression, and mortality in patients with OC. In addition, research studies and databases have demonstrated that both the expression and localization of thyroid hormone receptors alpha (TRα) and beta (TRβ) and membrane thyroid hormone receptor integrin alpha V beta 3 (αvβ3) affect OC progression and survival in OC patients. Furthermore, this review described the levels of the thyroid hormones (THs) thyroxine (T_4_) and 3,5,3′-triiodo-L-thyronine (T_3_) in the blood of OC patients and their role in OC progression. Moreover, we present studies that reported the relationship between hyperthyroidism and hypothyroidism and the levels of metabolic hormones in the blood and the possible effects on metabolic reprogramming in OC cells. We also report data indicating the relationship between the treatment of thyroid dysfunction and OC progression. Finally, the cited case studies described the essential case of struma ovarii, which is OC, including thyroid tissue. This review describes the link between thyroid dysfunction and OC risk and progression, which may be important in treating OC patients with thyroid dysfunction.

## 1. Introduction

The associations between thyroid disorders (hyperthyroidism and hypothyroidism) and hormone-dependent cancers have been highlighted in several epidemiological studies [1,2]. In particular, epidemiological data suggest a relationship between thyroid dysfunction and ovarian cancer (OC) [3,4,5]. In addition, abnormal levels of the thyroid hormones (THs) L-thyroxine (T_4_) and 3,5,3′-triiodo-L-thyronine (T_3_) may be responsible for the formation and progression of OC. Studies suggest that T_3_ and T_4_ promote OC development [6,7]. Furthermore, a significant positive or negative correlation has been identified between the survival of OC patients and the expression of nuclear thyroid hormone receptor alpha (TRα) and beta (TRβ) and the expression of the membrane thyroid hormone receptor integrin alpha V beta 3 (αvβ3). These studies suggest the potential role of thyroid hormone receptors in the adjuvant therapy of various cancers, including OC [8,9].

**Figure 1:**
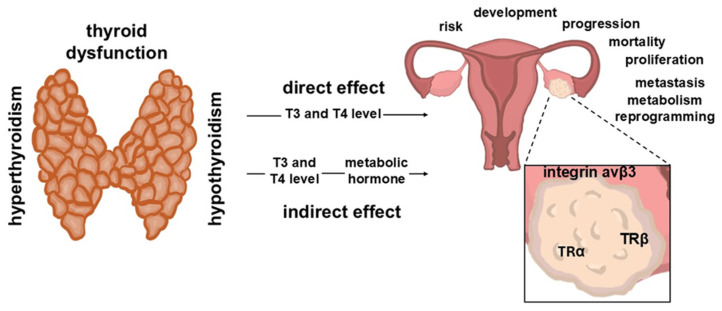
Direct and indirect effects of thyroid dysfunction (hyperthyroidism and hypothyroidism) via alterations in thyroid hormone levels (T_4_ and T_3_) on ovarian cancer (OC). T_4_—thyroxine; T_3_—triiodothyronine; TRα—thyroid hormone receptor α; TRβ—thyroid hormone receptor β.

This paper reviews the epidemiological, clinical, and experimental evidence for the link between thyroid dysfunction (hyperthyroidism and hypothyroidism) and OC, particularly concerning the possible molecular mechanisms underlying these associations. The potential role of hypothyroidism and hyperthyroidism in promoting OC is of particular interest (Figure 1). This review will also consider the impact of THs and their receptors on the progression of OC, including the effects on the genomic and nongenomic TH pathways. In addition, we present information on the indirect associations of thyroid dysfunction with OC progression and metabolism through the regulation of metabolic hormones. These publications indicate the dependence between the treatment of thyroid dysfunction and OC development. Finally, we describe a specific type of OC, namely, struma ovarii (SO).

## 2. Materials and Methods

### 2.1. Search Strategy

**Figure 2:**
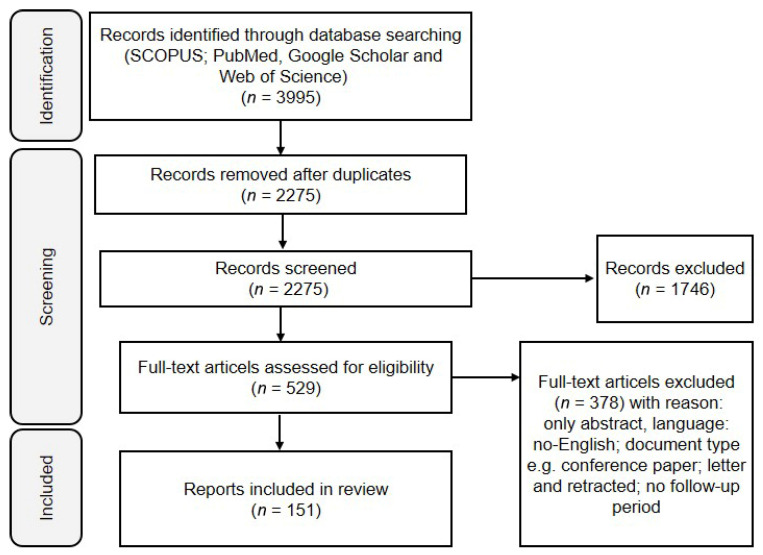
Flow chart review procedure.

The main focus of this review was the associations between thyroid dysfunction (hyperthyroidism and hypothyroidism) and OC risk, mortality, and progression. SCOPUS (https://www.scopus.com), PubMed (https://www.ncbi.nlm.nih.gov/pubmed), Google Scholar (https://scholar.google.com/), and Web of Science (https://www.webofscience.com) databases were searched on the basis of individual keywords and MeSH terms. Keywords were combined in various ways using Boolean operators ‘and’ or ‘or’ where appropriate and database-specific filters to maximize the identification of articles describing the association between thyroid dysfunction and OC. The search was performed, and all articles published up to March 2025 were retrieved. The articles included were clinical and epidemiological studies that reported the associations of the incidence of thyroid dysfunction with OC risk, mortality, and prognosis; studies that investigated the expression of nuclear and membrane TH receptors in OC; and studies that reported the level of TH in the blood of OC patients. There have also been studies that analyzed the effects of TH on OC progression and metabolism, studies that investigated the levels of metabolic hormones in OC, and analyses that explored the associations between thyroid dysfunction treatments and OC treatments. The search was limited to English literature, including meta-analyses, case reports, original studies, and reviews (Figure 2).

### 2.2. GEPIA Dataset Analysis

The GEPIA database [10] was used in this study to analyze the transcription levels and correlations of the *THRA*, *THRB*, *ITGAV*, and *ITGB3* genes in OC (*n* = 426) and normal (*n* = 88) tissues on 06.03.2025. Student’s *t* test was used to determine the *p* value. The *p* value cut-off was 0.05.

### 2.3. Kaplan–Meier Plot Analysis

The Kaplan–Meier plotter online global survival database (http://kmplot.com/analysis/), accessed on 1 March 2025 was used to evaluate the correlation between target gene mRNA expression and progression-free survival (PFS) and overall survival (OS) in the serous OC groups. The results are presented as Kaplan–Meier survival curves. The hazard ratio (HR) and 95% confidence interval (CI) were calculated automatically via the website tool. The values for each group are expressed as the means ± SDs. A log-rank *p* value < 0.05 was considered statistically significant [11].

## 3. Ovarian Cancer

### 3.1. Ovarian Cancer Epidemiology and Stages

According to the findings of the Centers for Disease Control and Prevention, ovarian cancer (OC) has been identified as the leading cause of death from gynecological malignancies on a global scale [12]. The Global Cancer Observatory, which covers 185 countries, reported that in 2022, the global incidence of OC was 324,603 cases, with a mortality rate of 206,956 [13,14,15]. Projections indicate that the estimated incidence of OC will reach approximately 477 thousand cases by the year 2045, with a corresponding mortality rate of approximately 328 thousand [15].

OC staging guidelines are determined by the International Federation of Gynecology and Obstetrics (FIGO) [16]. The survival probability is contingent upon the stage of the disease. For patients with early-stage disease (FIGO stage I or II), the five-year survival probabilities are 87% and 62%, respectively, whereas for patients with advanced-stage disease (FIGO stage III or IV), the probabilities are reduced to 26% and 14%, respectively [17]. The stage of OC is a critical factor in determining the most appropriate treatment and is also the most significant element in establishing prognosis. The high OC mortality rate is associated with nonspecific symptoms in the early stage, which means that 75% of women are diagnosed at an advanced stage [18].

### 3.2. Ovarian Cancer Classification

**Figure 3:**
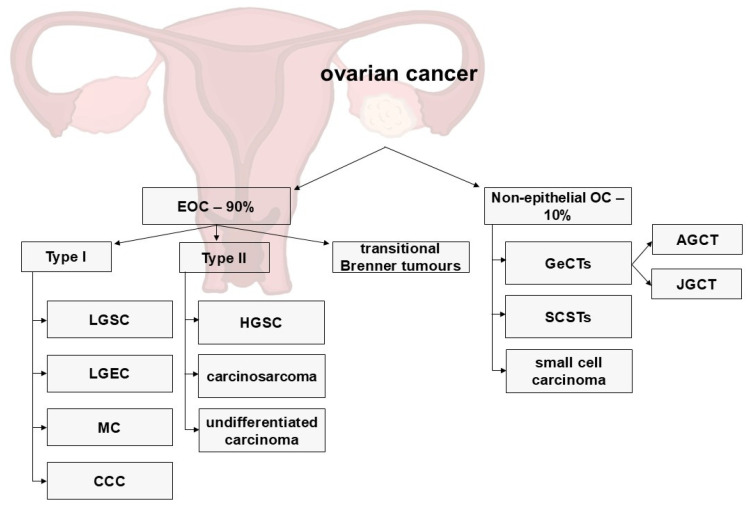
Classification of ovarian cancer (OC). Epithelial ovarian cancer (EOC) is divided into type I, type II and transitional Brenner tumors. Type I EOC includes low-grade serous carcinoma (LGSC), low-grade endometrioid carcinoma (LGEC), mucinous carcinoma (MC), and clear cell carcinoma (CCC). Type II EOC includes high-grade serous carcinoma (HGSC), carcinosarcoma, and undifferentiated carcinoma. Nonepithelial OCs include germ cell tumors (GeCTs), adult granulosa cell tumors (AGCTs), juvenile granulosa cell tumors (JGCTs), sex cord-stromal tumors (SCSTs) and rare tumors (small cell carcinomas).

OC is a heterogeneous tumor. Each histological subtype of OC has a different morphology, biological behavior, and prognosis. Ninety percent of OCs are epithelial OCs (EOCs), which originate from the epithelium [19]. The World Health Organization (WHO) divides EOCs into different groups on the basis of the type of cell: serous carcinoma (SC), mucinous carcinoma (MC), endometrioid carcinoma (EC), clear cell carcinoma (CCC), transitional cell, Brenner tumors, and mixed and undifferentiated. High-grade SC (HGSC) is the most common type of EOC [20]. Furthermore, recent molecular characterization of EOC correlated with clinicopathologic findings revealed that EOCs can be classified into two distinct groups. Type I (low-grade SC (LGSC), low-grade EC (LGEC), MC, and CCC) is relatively indolent and is diagnosed at an early stage, whereas type II (HGSC, undifferentiated carcinoma, and carcinosarcoma) is diagnosed at an advanced stage and has aggressive behavior, with a high rate of rapid dissemination [21]. Non-EOC comprises germ cell tumors (GeCTs), sex cord-stromal tumors (SCSTs), and rare tumors [22]. The most prevalent type of SCST is ovarian granulosa cell tumor (GCT), which accounts for approximately 5% of malignant OCs. GCTs can be categorized into two distinct subtypes, namely, adult-type GCTs (AGCTs) and juvenile GCTs (JGCTs), on the basis of their histological characteristics and clinical symptoms [23] (Figure 3).

## 4. The Associations of Hyperthyroidism and Hypothyroidism with Ovarian Cancer: Epidemiological, Clinical and Experimental Evidence

### 4.1. Thyroid Gland Dysfunction: Hyperthyroidism and Hypothyroidism

**Figure 4:**
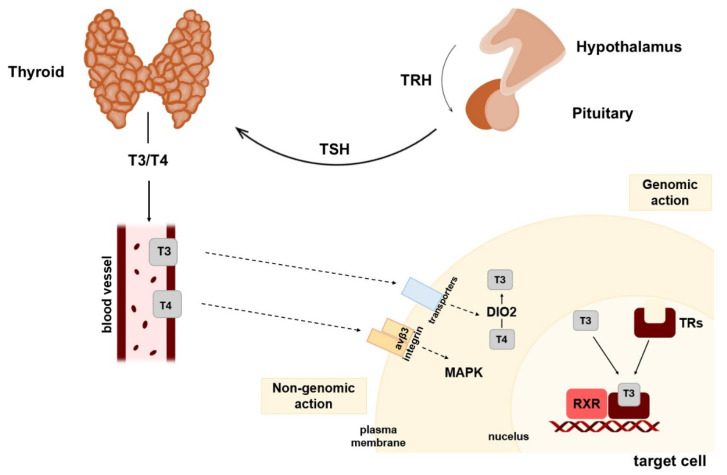
The regulation of thyroid hormone (TH) synthesis by the hypothalamus–pituitary–thyroid (HPT) axis and TH genomic and nongenomic action. TRH secreted by the hypothalamus stimulates the secretion of TSH by the pituitary. TSH stimulates the thyroid to synthesize and secrete T_4_ and T_3_ into the blood, which can enter target cells via transmembrane transporters. Inside the target cells, DIO2 converts T_4_ into T_3_. The classical genomic action of TH is mediated by binding within target cells to the nuclear TR subtypes TRα and TRβ. The nongenomic action of TH is associated with its action as a membrane thyroid hormone receptor integrin alpha V beta 3 (αvβ3) ligand, which activates specific signaling systems (trafficking and activation of mitogen-activated protein kinase (MAPK)). TH, thyroid hormone; TSH, thyroid-stimulating hormone; T_4_, thyroxine; T_3_, 3,3′,5′-triiodothyronine; HPT axis, hypothalamic–pituitary–thyroid axis; TRH, thyrotropin-releasing hormone; DIO, iodothyronine deiodinase; TRs, thyroid hormone receptors; integrin αvβ3 (integrin alpha V beta 3); MAPK, mitogen-activated protein kinase.

The thyroid is an endocrine organ responsible for the production and secretion of thyroid hormones (THs), including L-thyroxine (T_4_) and 3,5,3′-triiodo-L-thyronine (T_3_), which regulate metabolism and growth in organisms [24,25]. The synthesis and secretion of TH are regulated by the hypothalamus–pituitary–thyroid (HPT) axis [26,27] (Figure 4). The dysregulated production and secretion of TH by the thyroid gland are associated with the incidence of thyroid dysfunctions (hyperthyroidism or hypothyroidism). The prevalence is influenced by the iodine content of the diet [28,29].

Hyperthyroidism is characterized by increased TH synthesis and secretion from the thyroid gland, and the most common cause of hyperthyroidism is autoimmune inflammation of the thyroid gland, including Graves’ disease [30]. The prevalence of hyperthyroidism is approximately 0.8% in Europe and 1.3% in the USA [31,32]. The most prevalent etiological agent of hyperthyroidism is considered to be autoimmune inflammation of the thyroid gland [3]. Over the past decade, studies have indicated a correlation between elevated levels of inflammation and an increased risk of OC, decreased survival of patients with OC, and increased development of OC [33].

Hypothyroidism is characterized by a deficiency in TH. The condition is predominantly attributable to autoimmune thyroid disease, a subset of which is represented by Hashimoto thyroiditis [29]. The reported prevalence rates of hypothyroidism in Europe and the USA are 3% and 4.6%, respectively [30,31].

Thyroid dysfunctions are observed more frequently in women than in men [28]. There is a strong interaction between the HPT axis and the regulation of estradiol and progesterone secretion by the ovary [34]. Furthermore, hypothyroidism has been described as a potential causative agent in the formation of ovarian cysts [35]. In addition, Perveen et al. [36] described the association between hypothyroidism and multicystic ovarian enlargement in a 23-year-old woman in a case report.

### 4.2. The Prevalence of Thyroid Dysfunction and the Risk or Mortality of Ovarian Cancer

#### 4.2.1. Hyperthyroidism

In 2000, a population-based case–control study of OC (767 OC patients and 1367 controls) revealed that the risk of OC almost doubled in women aged 20–69 years with hyperthyroidism compared with women without a history of thyroid disease. These studies also suggest a potential link between hyperthyroidism and inflammatory responses within the ovarian epithelium [3]. A few years later, a potential association between hyperthyroidism and OC risk was reported in experimental studies [37]. A large study of a large group of people revealed that women with Graves’ disease have an increased risk of OC compared with women in the control group. This was even after the first year of follow-up had been terminated. The increased risk remained the same after a follow-up period of more than five years [38]. A recent study using two-sample Mendelian randomization analysis also revealed a correlation between an increased risk of OC and a genetic predisposition to hyperthyroidism. This finding suggests a possible causal relationship between hyperthyroidism and the development of OC [4]. Furthermore, this study demonstrated a potential link between thyroid dysfunction and increased mortality risk in OC patients. This assertion is supported by the findings of Journy et al. [5], who reported that OC patients diagnosed with thyroid dysfunction and treated with additional radioactive iodine presented a greater mortality risk than women with a healthy thyroid in the control group. In addition, studies have reported on a few cases where women with hyperthyroidism were at an elevated risk of OC mortality [5]. The Ovarian Cancer Association Consortium (OCAC) reported that a history of hyperthyroidism was linked to worse five-year survival. A diagnosis of hyperthyroidism within five years before OC diagnosis was associated with an increased risk of death; however, no significant relationship between hyperthyroidism history and OC mortality was observed [39]. A study conducted by Ramadan [40] reported that hyperthyroidism was significantly associated with an elevated risk of OC mortality.

In contrast, a cohort study of hyperthyroidism in relation to gynecological cancers revealed no significant association with the risk of OC. The present study sought to evaluate the link between self-reported physician-diagnosed hyperthyroidism in the Nurses’ Health Study (NHS) and the risk of medical record-confirmed OC [41]. In the cohort study by Leung et al. [42], the Taiwan National Health Insurance Research Database was utilized to investigate the correlation between hyperthyroidism and gynecological cancers in women from 2000 to 2018. Studies have indicated that women diagnosed with hyperthyroidism exhibit a significantly reduced risk of developing gynecological cancers, including endometrial cancer (EC), uterine corpus cancer (UC), and OC, irrespective of the specific cancer type [42]. Table 1 summarizes the effects of hyperthyroidism on OC risk and mortality. In summary, few studies have shown no correlation or a negative correlation between hyperthyroidism and the risk and mortality of OC, but the vast majority of studies have shown a positive association. Therefore, on the basis of the data presented here, we concluded that the prevalence of hyperthyroidism is related to an increased risk and mortality of OC.

#### 4.2.2. Hypothyroidism

Leung et al.’s cohort studies indicated that women with hypothyroidism presented a marginally elevated OC risk, a propensity that remained consistent irrespective of pharmaceutical interventions [42]. OCAC reported that an overall history of hypothyroidism was associated with worse five-year survival. A history of hypothyroidism was linked to a slightly increased risk of death [39].

Conversely, a study utilizing high-sensitivity chemiluminescence demonstrated that hypothyroidism does not impact the progression or prognosis of OC. The findings are somewhat contradictory, thereby hindering the ability to establish a definitive cause–effect relationship between thyroid dysfunction and OC [43]. A cohort study of hypothyroidism in relation to gynecological cancers also revealed no significant association with the risk of OC [41]. In addition, no such correlation was found between OC and hypothyroidism, the level of thyroid-stimulating hormone (TSH), or fT_4_ [4]. Furthermore, hypothyroidism has no statistically significant relationship with mortality in patients with OC [40]. The associations of hypothyroidism with OC risk and mortality are summarized in Table 1. There are fewer trials describing the relationships between hypothyroidism and OC risk and mortality, but most of them did not find a statistically significant association. On the basis of the small number of studies presented, it is not possible to establish a clear link between the incidence of hypothyroidism and OC, so further research and analysis are needed before a definitive conclusion can be reached.

### 4.3. Indirect Dependence of Thyroid Dysfunction and Ovarian Cancer

Furthermore, an indirect correlation between hypothyroidism and an elevated risk of OC has been indicated. The presence of hypothyroidism has been demonstrated to contribute to the development of polycystic ovary syndrome (PCOS), which affects ovulation and hormone balance [44]. This, in turn, has been linked to an increased risk of OC due to elevated androgen exposure [45]. According to a 2009 meta-analysis by Chittenden et al. [46], the incidence of OC is doubled in women with PCOS. Hansen et al. [47] reported a case of primary hypothyroidism with ovarian cyst formation and myxedematous infiltration of the ovary without luteinization.

## 5. The Expression Levels of Nuclear and Membrane Thyroid Hormone Receptors in Ovarian Cancer

### 5.1. The Expression of Nuclear Thyroid Hormone Receptors (TRs) in Ovarian Cancer: Classical Genomic Actions

THs bind with nuclear TR proteins, inducing transcription and activating or inhibiting various downstream effects on target genes [48]. Thyroid hormone receptors (TRs) belong to the nuclear receptor superfamily. Two TR proteins, TRα and TRβ, are encoded by two separate genes, *THRA* and *THRB* [49,50]. Distinct splicing forms of both TRα (TRα1, TRα2, and TRα3) and TRβ (TRβ1 and TRβ2) have been identified [51]. TRα1 is the sole protein that binds T_3_ and forms dimers with the truncated *THRA* gene products TRα2 and TRα3, which cannot bind this hormone [51]. However, both TRβ isoforms, TRβ1 and TRβ2, have been shown to bind T_3_ [52]. The binding of T_3_ to its receptors is critical for the biological effects of T_3_ across the body (Figure 4). TRα1 is present in many tissues, mainly in the heart, bones, and brain, whereas TRβ1 is more highly expressed in the liver, kidneys, and thyroid gland. In contrast, TRβ2 is found in the pituitary gland, hypothalamus, retina, and inner ear [53]. Since TRs have long been understood to function primarily by regulating gene transcription in the nucleus, their cytoplasmic localization could be interpreted as a regulator of their activity. Although TRs are generally considered to be predominantly located within the nucleus, TRα1 and TRβ1 have also been identified as capable of rapid shuttle movement between the nucleus and cytoplasm [54,55]. Furthermore, TR expression was observed in ovarian cells and OC tissue. As demonstrated by experimental studies, primary ovarian surface epithelial cells that potentially develop OC exhibit strong expression of TRs at the mRNA and protein levels [37,56].

**Figure 5:**
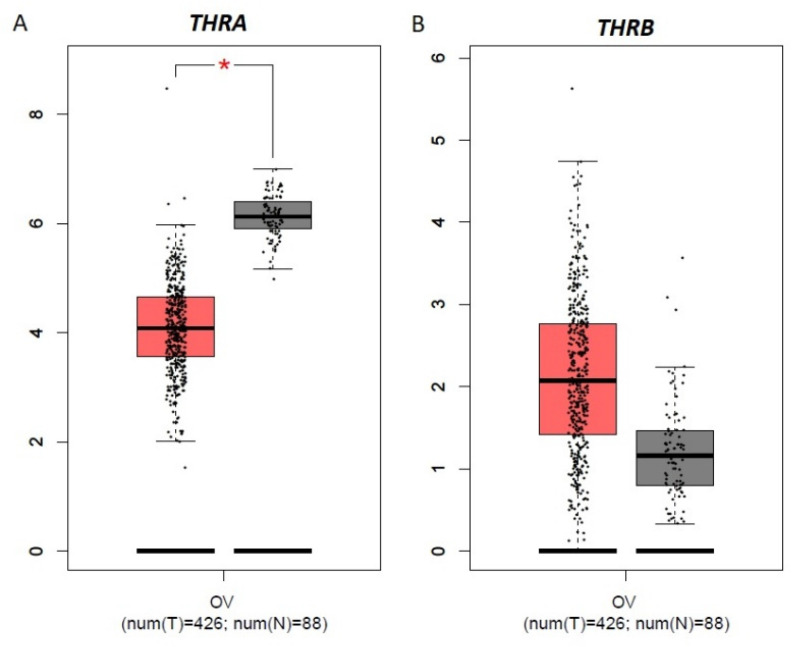
THRA and THRB expression in ovarian cancer (OC). The graphs of THRA (**A**) and THRB (**B**) mRNA expression generated from GEPIA ovarian tumors (T; in red; *n* = 426) compared with noncancer ovarian tissues (N; in gray; *n* =88). * *p* < 0.05.

According to the analysis via the GEPIA database, the expression level of *THRA* was significantly lower in OC than in noncancer ovarian tissue (Figure 5A; *p* < 0.05). However, the GEPIA database revealed no significant differences in the expression level of *THRB* between OC and noncancer ovarian tissues (Figure 5B). Importantly, these data were collected only for EOC, which accounts for the majority of OC cases, and did not consider the stage of the tumors.

Information on the expression levels of TRs (TRα and TRβ) in OC by type and stage is available from experimental literature data. Ditsch et al. [57] reported that among all OC subtypes, CC had the highest expression of TRα. Furthermore, nuclear TRα was associated with decreased survival in this subtype. There was a direct link between nuclear TRα1 or TRα2 expression and better survival in EOC patients, except in the CC subgroup. The group of OC patients with different CC subtypes seemed to have different characteristics in terms of TRα expression. In contrast, a shift in TRα1 or TRα2 expression to the cytoplasm seemed to be associated with reduced overall survival in EOC patients [57]. In the context of OC, both TRβ and its isoform TRβ1 are extensively expressed, with these proteins extending beyond the tumor cell nucleus to the cytoplasm. However, the subcellular distribution of these proteins varies among distinct histologic subtypes, grades, and FIGO stages. Notably, TRβ positivity has been strongly associated with decreased overall survival in OC patients. This correlation is attributed primarily to patients exhibiting complete or incomplete cytoplasmic shifts in TRβ. However, multivariate testing revealed that the prognostic significance of TRβ or its localization was lost [58]. Research has highlighted that the importance of TRs in terms of the progression and survival of OC patients is not contingent upon their level of expression but rather upon their localization.

**Figure 6:**
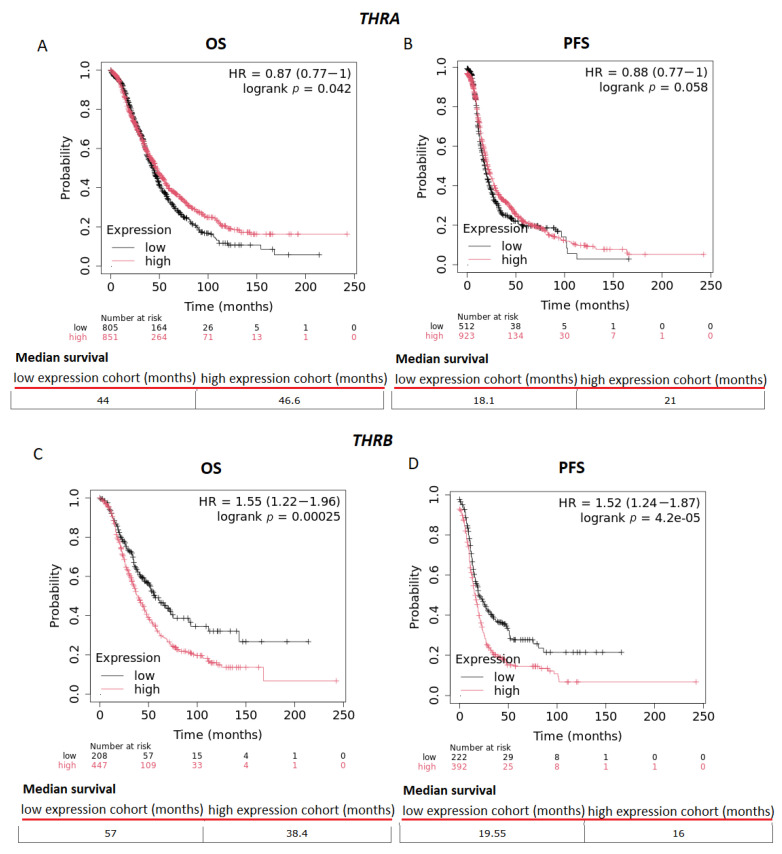
Thyroid receptor (THRA and THRB) expression levels and survival in ovarian cancer (OC) patients. Kaplan–Meier survival plots of patients with OC. Overall survival (OS) and progression-free survival (PFS) associated with the THRA expression level (**A**,**B**) and THRB expression level (**C**,**D**) are shown. The probe IDs are as follows: THRA (1316_at, 204100_at, 204760_s_at, 214883_at, 217476_at, 31637_s_at 35846_at) and THRB (207044_at, 228716_at, 229657_at). The hazard ratio (HR) is indicated, along with the 95% confidence interval (CI). The log-rank P test is displayed at the upper right corner of every graph [11].

However, Kaplan–Meier survival analysis revealed better overall survival (OS) and progression-free survival (PFS) in all OC patients of all stages and grades with high *THRA* expression and worse OS and PFS in all OC patients with high *THRB* expression. The risk of death is decreased by approximately 13% in OC patients with high *THRA* expression and increased by approximately 55% in OC patients with high *THRB* expression. The median survival of OC patients with low *THRA* expression was estimated to be 44 months, and that of OC patients with high THRA expression was 46.6 months. The median survival of OC patients with low *THRB* expression was estimated to be 57 months, and that of OC patients with high THRB expression was 38.4 months (Figure 6A,C; *p* = 0.042 and *p* < 0.001, respectively). Furthermore, PFS was decreased by approximately 52% in OC patients with high *THRB* expression (median survival: 19.55 months for OC patients with low *THRB* expression and 16 months for OC patients with high *THRB* expression) (Figure 6C,D; *p* = 0.058 and *p* < 0.001, respectively).

The available studies do not allow definitive conclusions to be drawn. However, in summary, the database revealed a correlation between poor overall survival in OC patients with low *THRB* expression and high *THRA* expression. In addition, research has shown that the level of TRα and TRβ expression and the correlation of TR expression with survival in OC patients are dependent on tumor type and stage. These studies highlighted that the subcellular localization of TRs, particularly the transition from nuclear to cytoplasmic localization, is a more significant predictor of survival in patients with OC than the level of TR expression. However, further studies are needed to support this.

### 5.2. Integrin αvβ3 Expression in Ovarian Cancer: Nongenomic Mechanism of Action

As demonstrated in the study by Bergh et al. [59], nongenomic actions of TH (T_3_ and T_4_) have been shown to bind to integrin αvβ3, which is a structural protein located within the plasma membrane, thereby activating protein kinase/extracellular signal-regulated kinase pathways [59,60] (Figure 4). The integrin αvβ3, also known as the vitronectin receptor, consists of a 125 kDa αv subunit (encoded by the *ITGAV* gene) and a 105 kDa β3 subunit (encoded by the *ITGB3* gene) [61]. The integrin αvβ3 pathway is critical for regulating processes such as cell proliferation, apoptosis, and metastasis in cancer cells [62].

**Figure 7:**
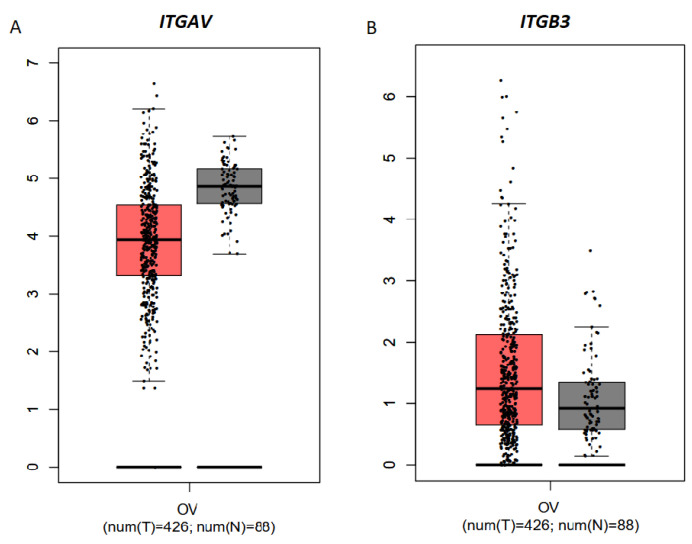
Expression of two subunits of integrin αvβ3 in ovarian cancer (OC). Graphs of ITGAV (**A**) and ITGB3 (**B**) mRNA expression in GEPIA-identified ovarian tumors (T; red, *n* = 426) compared with normal tissues (N; gray, *n* =88).

GEPIA database analysis of 426 samples of EOC and 88 noncancer ovarian tissues, without considering the stage of the tumor, revealed that the expression levels of *ITGAV* and *ITGB3* were not significantly different between OC and noncancer ovarian tissues (Figure 7A).

However, experimental data have provided evidence both for the current expression of integrin αvβ3 and for its role in the progression of OC. In their 2020 study, Seraya-Bareket et al. [63] reported the presence of the nuclear integrin αvβ3 in HGSC cells and tissues but not in normal ovaries or fallopian tubes (FTs). The nuclear integrin αvβ3 was found to be functionally active. This study demonstrated that OVCAR-3 cells with high expression of nuclear integrin αvβ3 exhibited increased proliferation and oncogenic signaling, inhibiting migration and intact colony formation ability. Proteomic analyses revealed a network of nuclear integrin αvβ3-bound proteins, many of which have key cancer-related activities. As Shinderman-Maman et al. [6] demonstrated, T_3_ has been shown to bind to the integrin αvβ3, which is overexpressed in OC cells. The OVCAR-3 cell line, which represents HGSC (type II EOC), presented the highest level of integrin αvβ3. In contrast, LGSC (type I EOCs (SKOV-3 and A2780 cell lines)) presented lower expression of integrin αvβ3. These findings suggest that higher levels of integrin expression are observed in late-stage disease [6]. Furthermore, the expression of integrin αvβ3 is less common in EOCs with low malignant potential, which is characterized by a histological absence of invasion and a very good prognosis. This is in contrast to invasive OC [64]. As Wang et al. [65] demonstrated, the expression rate of integrin αvβ3 in malignant EOC was significantly greater than that in the borderline benign group and the normal ovary group. In addition, T_4_-induced nuclear accumulation of the nuclear αvβ3 integrin transcription complex enhances OC proliferation [66]. The EOC integrin αvβ3-transfected cell model used by Dolinschek et al. [67] demonstrated the ability of integrin αvβ3 to promote the activation of key players involved in anoikis resistance, including epidermal growth factor receptor (EGF-R), mitogen-activated protein kinase (MAPK), focal adhesion kinase (FAK), src, and PKB/Akt kinases, as well as the induction of antiapoptotic factors. Furthermore, research findings have demonstrated that the interaction between integrin αvβ3 plays a pivotal role in stimulating the proliferation and motility of OC cells (OV-MZ-6) [68]. Lössner et al.’s [69] findings suggest that the upregulation of EGF-R expression and activity in human OCs is mediated by integrin αvβ3. In addition, Gao et al. [70] reported a close correlation between integrin αvβ3 and Lewis and antigen expression, indicating that there are independent drug resistance-related risk factors in patients with OC. In contrast, one study by Kaur et al. [71] revealed that the inhibition of integrin αvβ3 in OC cells increased proliferation and invasion but decreased adhesion. OC patients with high tumor expression of integrin αvβ3 had significantly better overall survival, suggesting that integrin αvβ3 acts as a tumor suppressor in OC cells [71].

**Figure 8:**
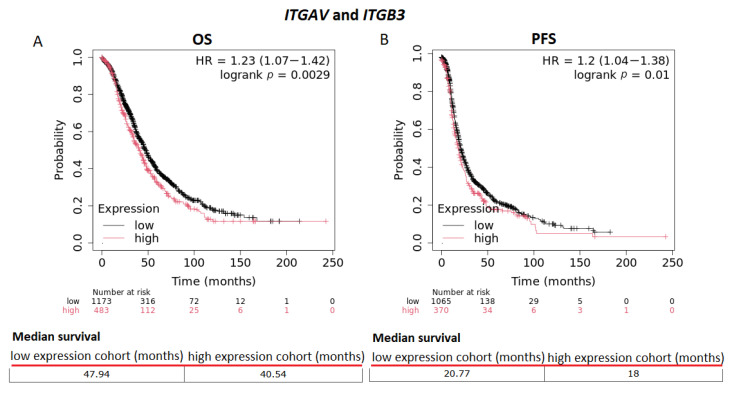
Integrin αvβ3 (subunits: ITGAV and ITGB3) expression levels and survival in ovarian cancer (OC) patients. Kaplan–Meier survival plots of patients with OC. (**A**) Overall survival (OS) and (**B**) progression-free survival (PFS) are shown; the probe IDs are as follows: ITGAV (202351_at) and ITGB3 (204625_s_at, 204626_s_at, 204628_s_at, 215240_at, 216261_at, 204627_s_at, 211579_at). The hazard ratio (HR) is indicated, along with the 95% confidence interval (CI). The log-rank P test is displayed at the upper right corner of every graph [11].

The Kaplan–Meier survival analysis revealed worse overall survival (OS) and progression-free survival (PFS) in all OC patients of all stages and grades with high mean expression of *ITGAV* and *ITGB3.* The risk of death is increased by approximately 23% in OC patients with high mean expression of *IGAV* and *ITGB3*. The median survival of OC patients with low mean expression of *ITGAV* and *ITGB3* was estimated to be 47.94 months, and that of OC patients with high mean expression of *ITGAV* and *ITGB3* was 40.54 months. Progression-free survival was reduced by approximately 20% (median survival: 20.77 months for OC patients with low mean expression of *ITGAV* and *ITGB3* and 18 months for OC patients with high mean expression of *ITGAV* and *ITGB3)* (Figure 8A,B; *p* = 0.0029 and *p* = 0.01, respectively).

In conclusion, studies indicate that integrin αvβ3 is overexpressed in OC, highlighting differences in its expression levels and localization depending on the OC type and disease stage. Moreover, databases have indicated that high expression levels of integrin αvβ3 in OC patients are correlated with poor overall survival and progression-free survival. Furthermore, on the basis of the numerous studies presented, we conclude that integrin αvβ3 plays a role in OC proliferation and metastasis.

## 6. Relationship Between Thyroid Hormone Levels and the Progression of Ovarian Cancer

### 6.1. Thyroid Hormone Levels in Ovarian Cancer Patients

THs play an important role in several key processes, including growth, development, tissue homeostasis, and cancer [48]. Given the established relationship between thyroid disorders and alterations in TH expression on one hand, and ovulation and estrogen levels on the other hand, further exploration into the possible association with OC is needed.

A study by Rasool et al. [72] revealed elevated levels of T_4_, but no statistically significant differences were observed in the levels of T_3_ in the serum samples compared with those in the control group. However, the TH levels in patients with OC deviated from the standard reference ranges compared with those observed in healthy individuals [72]. Studies conducted on samples from 100 healthy women and 144 women with diagnosed gynecological cancer (128 cases of OC and 16 cases of endometrioid cancer (EC)) indicated a deranged thyroid profile with gynecological cancer. Elevated serum TSH levels are associated with the occurrence of OC and EC [73].

Correlation analysis revealed abnormal levels of TSH, fT_3_, fT_4_, and thyroid-stimulating hormone receptor (TRAb) antibodies in the serum of OC patients. The positive rates of TSH, fT_3_, fT_4_, and TRAb varied according to the type of OC (serous cystadenocarcinoma showed higher rates than did mucinous cystadenocarcinoma and other types), stage (lower rates in stages III and IV than in stages I and II), and differentiation (higher rates in poorly differentiated OC than in moderately differentiated and well-differentiated OC compared with the control group). In addition, significant differences in the serum levels of TSH, fT_3_, fT_4_ and TRAb were detected compared with those in the control group, both before and after surgery and after four cycles of OC [74].

In conclusion, determining a direction of change on the basis of these data is difficult, although several meta-analyses have shown abnormalities in the TH profile of patients with OC.

### 6.2. The Role of Thyroid Hormones in Ovarian Cancer Progression

**Figure 9:**
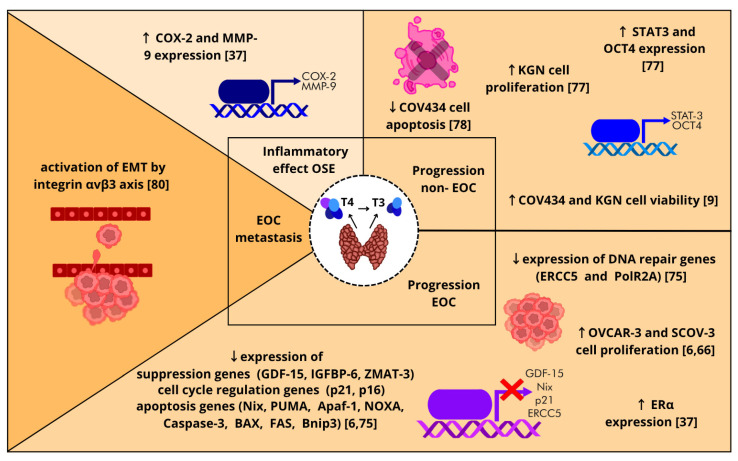
Schematic representation of the possible actions of thyroid hormones (THs) in the ovarian surface epithelium (OSE) and ovarian cancer (OC). T_3_ exerts direct inflammatory effects on OSE. THs increase OC progression (both nonepithelial OC and epithelial OC (EOC)) by regulating the proliferation and expression of genes associated with tumor suppression, apoptosis, DNA repair, and cell cycle regulation. T_3_ promotes OC metastasis by enhancing epithelial-to-mesenchymal transition (EMT). ↑—significant increase, ↓— significant decrease; COX-2—cyclooxygenase-2; MMP-9—matrix metalloproteinase-9; STAT3—signal transducer and activator of transcription 3; OCT4—octamer-binding transcription factor 4; ERCC5—gene encoding the DNA repair protein complementing XP-G cells; PolR2A—gene encoding the DNA-directed RNA polymerase II subunit RPB1; Erα—estrogen receptor alpha; GDF-15—growth/differentiation factor 15; IGFBP-6—insulin-like growth factor-binding protein 6; ZMAT-3—zinc finger matrin-type 3; p21—cyclin-dependent kinase inhibitor 1; p16—cyclin-dependent kinase inhibitor 2A; Nix—membrane-anchored outer mitochondrial protein; PUMA—p53 upregulated modulator of apoptosis; Apaf-1—apoptotic protease activating factor 1; NOXA—phorbol-12-myristate-13-acetate-induced protein 1; BAX—bcl-2-like protein 4; FAS—cell surface death receptor; Bnip3—BCL2 interacting protein 3; COV434—juvenile granulosa tumor cell line; KGN—adult granulosa tumor cell line; OVCAR-3 and SKOV-3—epithelial OC (EOC) cell lines [6,9,37,66,75,77,78,80].

T_3_ exerts direct inflammatory effects on ovarian surface epithelial (OSE) cell function in vitro, with the potential to affect the tumor microenvironment. As shown in in vitro studies, T_3_ affects OSE cells by inducing inflammation in OSE cells and increasing the expression of inflammation-associated genes such as cyclooxygenase-2 (COX-2) and matrix metalloproteinase-9 (MMP-9). In addition, T_3_ stimulates the expression of estrogen receptor alpha (ERα) mRNA, which is strongly associated with OC progression [37] (Figure 9).

Recent findings have indicated a potential correlation between exposure to THs and the development and progression of OC. An in vitro study demonstrated that physiological levels of T_3_ (1 nM) and T_4_ (100 nM) can induce MAPK-dependent proliferation and increase the survival of OC cells (OVCAR-3) after three- and five-day incubation. Furthermore, in A2780 cells, longer incubation with either hormone (five days) was needed to observe the effect on A2780 cell viability. Conversely, exposure to these hormones at concentrations below normal serum levels may reduce the growth of OC cells by approximately 10% (OVCAR-3). Additionally, the inhibitory effects of THs on the expression of six genes associated with tumor suppression (GDF-15, IGFBP-6), apoptosis (Nix, PUMA), and cell cycle regulation (p21, p16) were documented in OC cell lines [6]. Two years later, Shinderman-Maman et al. [75] reported that THs can downregulate the expression of apoptosis-related genes (*Apaf-1*, *NOXA*, *Caspase-3*, *BAX*, *FAS*, *Bnip3*), a tumor suppressor gene (*ZMAT-3*) and two DNA repair genes (*ERCC5* and *PolR2A*). This finding highlights the role of THs in influencing the molecular pathways related to OC progression [75]. In addition, Shinderman-Maman et al. [76] reported that derivatives of TH, including tetrac, triac, and T1AM, have the capacity to reduce proliferation and induce cell death and DNA damage in the OC cell lines OVCAR-3 and A2780. This finding suggests a potential therapeutic approach for OC [76]. Furthermore, studies by Hsieh et al. [66] reported the proliferative effects of THs (100 nM T_4_ and 10 nM T_3_) on OC cells (OVCAR-3 and SKOV-3). These processes are initiated via integrin αvβ3, which has the potential for subsequent crosstalk with ERα. The activation of ERK1/2 by THs was found to be integrin αvβ3-dependent. Furthermore, it was determined that activated ERK1/2 was responsible for the phosphorylation of ERα. The collaboration between these two signaling pathways is pivotal in promoting OC cell proliferation. The findings of this study provide a novel direction for the management of ERα-positive OC, with a focus on the role of THs in the absence of host estrogen [66] (Figure 9).

Yu et al. [77] reported that T_3_ increases cell viability and proliferation in ovarian adult granulosa cell tumors (KGN cell line). This effect is mediated by the TR and involves the phosphorylation of signal transducer and activator of transcription (STAT3) and the expression of octamer-binding transcription factor 4 (OCT4) proteins. Blocking TR or inhibiting STAT3 reduces T_3_-induced cell growth, suggesting that T_3_ promotes cell development via the STAT3-OCT4 pathway, which is associated with poor overall survival in OC patients [77]. Our previous studies demonstrated that T_3_, in a dose-dependent manner ranging from 0.1 nM to 100 nM, increased the viability of nonluteinized ovarian granulosa cells (HGrC1) and rare OC cells (COV434 and KGN cells). This effect was observed in comparison with that in untreated control cells [9]. A report by Verga Falzacappa et al. [78] indicated that T_3_, but not T_4_, is capable of protecting COV434 cells from apoptosis. This protective effect is achieved through the regulation of the cell cycle and cell growth. Moreover, the protective effect of T_3_, as evaluated by FACS analysis in the presence of a PI3K inhibitor, is significant. This finding was further substantiated by Western blot analysis of pAkt, which confirmed the crucial role of the PI3K pathway in T_3_ survival [78] (Figure 9).

Furthermore, T_3_ can enhance angiogenesis and tumor metastasis [79]. In OC, T_3_ through the integrin αvβ3 pathway is involved in epithelial-to-mesenchymal transition (EMT) activity. This finding highlights a novel role for THs in OC metastasis [80] (Figure 9). In addition, T_4_ inhibits resveratrol-induced apoptosis via programmed death-ligand 1 (PD-L1) in in vitro model OC cells. The inhibition of resveratrol-induced nuclear accumulation of COX-2 and increased expression and cytoplasmic accumulation of PD-L1 were observed after treatment with T_4_ [81].

In summary, the available information from in vitro studies strongly suggests that THs may influence increased inflammation in OSE as well as OC progression and metastasis. To date, the molecular mechanism by which THs promote OC proliferation and inhibit OC apoptosis by regulating the expression of suppressive tumor genes, cell cycle regulatory genes, apoptosis genes, and DNA repair genes has been described.

### 6.3. The Role of Iodothyronine Deiodinases in Ovarian Cancer Progression

TH deiodinases play pivotal roles in various physiological processes, including development, growth, and metabolic regulation. These enzymes function as regulatory agents, maintaining the balance of THs within the body. To date, three distinct deiodinases have been identified: iodothyronine deiodinase 1 (DIO1), DIO2, and DIO3 [82]. DIO1 and DIO2 convert T_4_ or reverse T_3_ (rT_3_) to T_3_ [83] (Figure 4).

As Moskovich et al. [84] reported, an increase in T_3_ bioavailability through stable knockdown of DIO3 (DIO3-KD) resulted in the induction of apoptosis and the attenuation of proliferation, migration, colony formation, oncogenic signaling, and tumor growth in a mouse model of HGSC. Lower levels of DIO1 were observed in HGSC tissues than in normal tissues. TCGA analyses confirmed that low DIO1 mRNA expression was correlated with poorer survival and resistance to therapy in HGSC patients. Silencing or inhibiting this enzyme resulted in increased HGSC proliferation. These studies showed that DIO1 has a potential role in suppressing OC [85]. However, DIO2 has been shown to act as a tumor promoter in OC and is proposed as an oncogenic marker [86].

## 7. Thyroid Dysfunctions and Ovarian Cancer Metabolism

There is evidence that THs have a strong effect on glucose metabolism, which was reported more than 100 years ago [87]. The uninterrupted proliferation that is characteristic of cancer, including OC, is connected with a phenomenon termed the “Warburg effect”. This effect refers predominantly to the production of energy through aerobic glycolysis rather than mitochondrial respiration [88]. The associations of metabolic reprogramming and the Warburg effect with THs in a variety of cancers, including breast cancer and renal cell carcinoma, have been described in in vitro studies [89,90].

Only four studies have reported the role of THs in OC metabolism. Studies suggest that T_3_ plays a role in the PI3K/AKT/mTOR pathway, which is frequently altered in OC and is involved in cellular metabolism and tumor progression [37,91]. In vivo studies revealed that stable DIO3 knockdown, which led to increased T_3_ bioavailability in HGSC cells, promoted metabolic reprogramming by inducing the Warburg effect in a HGSC mouse model [84]. In our recent study, no alteration in the metabolic activity of adult granulosa cell tumor (KGN) cells was detected after treatment with T_3_ (10 nM). However, a decrease in metabolic activity was observed following treatment with GW0742 (a potential novel TRα and TRβ antagonist) at a dose of 10 μM. The findings indicated a decrease in total ATP levels, accompanied by a decrease in glycoATP and mitoATP production rates, as well as an alteration in the oxygen consumption rate (OCR) and the extracellular acidification rate (ECAR). These observations suggest a substantial reduction in the mitochondrial and glycolytic pathways in KGN cells [9].

In summary, despite the potential role of THs in the regulation of OC metabolism, there is an emerging knowledge gap. This is due to the lack of research on this topic.

### 7.1. Associations Between Thyroid Dysfunction and the Blood Levels of Metabolic Hormones, Including Adipokines

Single cells or spheroids of OC cells are capable of surviving in nonadherent conditions, as evidenced by their resistance to anoikis (a specific form of apoptosis triggered by detachment from the extracellular matrix) [92,93]. This process is closely linked to the regulation of metabolic reprogramming, which can be regulated by individual metabolic hormones, including adipokines, which are released by adipose tissue [94,95]. The detection of adipokines (e.g., visfatin, adiponectin, and leptin) in the peritoneal fluid of OC patients suggests that they may act on OC cells, which have receptors for certain metabolic hormones on their surface [96,97,98]. Therefore, as key mediators of metabolic regulation, they can alter OC metabolism via paracrine signaling [99]. The impact of THs on metabolic hormone production has received little attention from the scientific community.

#### 7.1.1. Insulin

The Mendelian randomization data revealed that an increased risk of OC is associated with a decreased rate of insulin secretion. These findings suggest that glucose metabolism, which is regulated by insulin secretion, plays an important role in the pathogenesis of OC, which involves the expression of a functional insulin receptor [100,101]. Furthermore, Joung et al. [102] reported that hyperinsulinemia is linked with an elevated risk of OC in women after menopause.

THs have been shown to induce hyperinsulinemia and stimulate insulin secretion [103,104]. Yang et al. [105] reported that subclinical hypothyroidism is connected with increased hyperinsulinemia. In addition, the half-life of insulin is reduced by an increased rate of degradation in diabetic patients with hyperthyroidism [106]. Furthermore, insulin resistance has been described as a result of thyroid dysfunction, both hyperthyroidism and hypothyroidism. In hyperthyroidism, an imbalance of THs can lead to glucose intolerance, mainly due to hepatic insulin resistance [107]. Another study confirmed that hypothyroidism leads to decreased synthesis and release of insulin [108] and insulin resistance [109]. Moreover, insulin also regulates TH levels by increasing fT_4_ while suppressing T_3_ levels by inhibiting the conversion of T_4_ to T_3_ [110] (Table 2).

#### 7.1.2. Adiponectin and Leptin

The roles of adiponectin and leptin in OC have been linked. The expression of adiponectin (AdipoR1) and leptin receptor was detected in OC cells [97,98]. In a seminal study, Diaz et al. [111] demonstrated that a high leptin/adiponectin ratio was correlated with a poor prognosis for patients diagnosed with OC.

The study revealed higher concentrations of serum adiponectin in patients with hyperthyroidism than in patients with hypothyroidism (5.73 vs. 3.0 ng/mL) and revealed a positive correlation between adiponectin levels and fT_4_ and fT_3_ [112]. The levels of leptin in the serum did not change with thyroid functional status [112] (Table 2).

#### 7.1.3. Apelin

The presence of apelin and its secretion at low concentrations (<0.3 ng/mL) has been identified in HGSC [113,114]. Moreover, apelin and its receptor (APJ) are expressed in various OC cell lines [115]. Research has revealed that apelin promotes the progression and metastasis of OC by regulating energy metabolism and lymphatic vessel formation in serous OC [116]. Furthermore, human OC cells with high APJ expression presented significant increases in migration and invasion in vitro [117].

The results revealed that the serum levels of apelin were increased in hypothyroid rats. In addition, apelin in the presence or absence of THs decreases serum levels of TSH, but lowering TSH is more effective in the coadministration of T_4_ [118,119]. Analysis of apelin levels in different TH states revealed that apelin levels were higher in patients with hypothyroidism (4.8 ± 2.5 ng/mL) and hyperthyroidism (3.7 ± 1.9 ng/mL) than in the normal population (3.4 ± 1.4 ng/mL), but the difference was not statistically significant [120]. Zorlu et al. [121] reported that apelin levels did not differ between patients with subclinical hypothyroidism and healthy subjects (Table 2).

#### 7.1.4. Chemerin

Chemerin was found to be abundant in the ascitic fluid of OC patients [122]. Furthermore, chemerin expression is higher in OC tissue than in noncancer ovarian tissue [123], and the chemerin receptor chemokine-like receptor 1 (CMKLR1) is expressed in OC cells [124]. The biological impact of chemerin on signaling pathways in OC has been described [125,126].

Serum chemerin levels were found to be significantly elevated by approximately 13 µg/L in patients diagnosed with hyperthyroidism compared with those in the control group (88 vs. 75 µg/L, respectively). Furthermore, a positive correlation was detected between serum chemerin concentrations and T_3_, T_4_, and fT_3_, whereas a negative correlation was detected between chemerin levels and TSH and FT_4_ [127] (Table 2).

#### 7.1.5. Resistin

Studies by Pang and Chang [128] indicated that resistin can promote the proliferation and migration of OC cells.

A meta-analysis of the extant literature demonstrated that resistin levels are significantly higher in patients with thyroid dysfunction. This finding suggests a potential association between higher TH levels and increased resistin. Moreover, a significant decrease in resistin levels after treatment in patients with thyroid dysfunction was observed in comparison to levels before treatment [129]. As demonstrated in the literature, serum resistin concentrations were found to be higher in hyperthyroidism patients than in hypothyroidism patients (6.378 vs. 5.81 ng/mL, respectively) [112]. Additionally, the successful treatment of hyperthyroidism is associated with decreased resistin levels [130]. As demonstrated in the study by Eke Koyuncu et al. [131], an increase in resistin levels was found to be directly related to thyroid dysfunction. The levels of resistin in the hypothyroid group were significantly greater than those in the control group (12.66 and 8.45 ng/mL, respectively). Similarly, resistin levels were significantly higher in the subclinical hyperthyroid group than in the control group (14.88 and 8.45 ng/mL, respectively). Furthermore, changes in TH levels affect the synthesis and/or secretion of resistin in adipose tissue and/or macrophages [131]. Additionally, a positive correlation between resistin levels and fT_3_ levels in patients with thyroid dysfunction was identified [129,130]. Moreover, a positive correlation between resistin levels and fT_4_ levels, as well as a negative correlation with TSH, was observed [130].

Studies conducted by Owecki et al. [132] demonstrated that short-term profound hypothyroidism resulted in a decrease in resistin levels. However, no significant correlations were detected between the levels of circulating resistin and THs or TSH [132]. In addition, Peng et al. [133] reported that downregulated resistin levels in Grave’s disease patients are the consequence of decreased neutrophil counts (Table 2).

#### 7.1.6. Visfatin (NAMPT)

Li et al. [96] reported that visfatin is present in the ascitic fluid of OC patients. The increased expression of visfatin is associated with OC progression through the upregulation of pro-proliferative cytokine secretion and the stimulation of OC growth, development, angiogenesis, and metastasis [96,134,135].

Ozkaya et al. [136] suggested that serum visfatin levels in a cohort of patients with hyperthyroidism and hypothyroidism may be modulated by THs, such as T_3_ and T_4_. Low visfatin levels were detected in patients with hyperthyroidism (9.44 ng/mL) compared with those in patients with hypothyroidism (49.93 ng/mL) and the control group (38.6 ng/mL). In patients diagnosed with hypothyroidism, a significant decrease in plasma visfatin levels was observed following treatment (58.58 ng/mL vs. 40.00 ng/mL). Furthermore, a negative correlation was identified between visfatin levels and both fT_3_ and fT_4_ levels, whereas a positive correlation was detected between visfatin and TSH levels. Moreover, visfatin, as a potential proinflammatory mediator, may contribute to proinflammatory conditions in the thyroid, leading to thyroid dysfunction [136]. Serum visfatin concentrations were found to be significantly lower in patients with hyperthyroidism than in controls. Furthermore, a negative correlation was identified between fT_4_ and T_4_ concentrations, on the one hand, and serum visfatin concentrations, on the other hand. Conversely, TSH was found to correlate positively with serum visfatin levels [127] (Table 2).

#### 7.1.7. Vaspin

As González et al. [137] demonstrated, thyroid dysfunction has the potential to affect vaspin expression. Researchers have reported that the mRNA levels of vaspin are considerably lower in hyperthyroid rats and significantly greater in hypothyroid rats than in euthyroid rats [137]. Handisurya et al. [138] reported a significant decrease in TSH levels that was positively correlated with changes in serum vaspin levels. In contrast, a study by Cinar et al. [139] demonstrated that TH status does not influence serum vaspin levels in humans. Vaspin levels were similar in euthyroid and hypothyroid patients. However, vaspin levels do not correlate with TSH levels [139] (Table 2).

#### 7.1.8. Omentin (ITLN1, Intelectin-1)

In patients diagnosed with neoplasia, omentin levels are lower than those observed in patients with benign gynecological lesions and healthy women. Furthermore, the downregulation of mesothelial cell-derived ITLN1 in the omental tumor microenvironment has been demonstrated to facilitate OC progression [140].

As Alshaikh et al. [127] reported, serum omentin concentrations were significantly lower in patients with hyperthyroidism than in the control group (~30 vs. ~70 µg/L). Cerit et al. [141] reported that, in hypothyroid patients, omentin-1 levels were lower than those in the control group but increased after six months of TH replacement therapy. However, no significant correlation was observed between serum omentin concentrations and any of the thyroid profile variables except fT_3_ [127] (Table 2).

**Table 2:** The blood levels of metabolic hormones, including adipokines (insulin, adiponectin, leptin, apelin, chemerin, resistin, visfatin, vaspin, and omentin), in patients with thyroid dysfunction (hyperthyroidism and hypothyroidism) and correlations between thyroid and metabolic hormones.

Metabolic Hormones	Blood Levels of Metabolic Hormones in Patients with Thyroid Dysfunction Compared with Control Subjects			Correlation Between Metabolic Hormone Level and TH Levels	
	Hyperthyroidism	Hypothyroidism		Positive	Negative
**insulin**	reduced half-life by an increased rate of degradation in diabetic patients [106]	associated with increased hyperinsulinemia [105]		fT_4_ [110]	T_3_ [110]
	insulin resistance—result of both thyroid dysfunctions [107]			THs induce hyperinsulinemia and stimulate insulin secretion [103,104]	
**adiponectin**	↑ (5.73 ng/mL) [112]	↓ (3 ng/mL) [112]		fT_3_, fT_4_ level [112]	Nd.
**leptin**	no change detected [112]			Nd.	
**apelin**	↓ (3.7 ± 1.9 ng/mL) [120]	↑ 4.8 ± 2.5 ng/mL) [120]		Nd.	↓ TSH levels (with a stronger effect when coadministered with T_4_) [118,119]
	normal population (3.4 ± 1.4 ng/mL) [120] apelin levels remain unchanged in subclinical hypothyroidism [121]				
**chemerin**	↑ 88 µg/L [127]	Nd.		TT_3_, TT_4_ and fT_3_ levels [127]	TSH and fT_4_ levels [127]
**resistin**	↑ 6.378 ng/mL [112] ↑ 14.88 ng/mL [131] control (8.45 ng/mL) [131]	↑ 5.81 ng/mL [112] ↑ 12.66 ng/mL [131] control (8.45 ng/mL) [131]		fT_3_ [129,130] fT_4_ [130] successful treatment of hyperthyroidism associated with decreased resistin levels [112,130]	TSH [130] short-term profound hypothyroidism decreases resistin level [132]
	↓ in Graves’ disease, as a result of a decrease in the number of neutrophils [133]			no significant correlations between TH or TSH levels [131]	
**visfatin**	↓ (9.44 ng/mL) [136]	↑ 49.93 (ng/mL) [136]		TSH [127,136]	fT_3_ [136] fT_4_ and TT_4_ [127,136]
	control—38.6 ng/mL [136]			visfatin levels is decreased by T_3_ and T_4_ [112]	
**vaspin**	Nd.	overt 1.20 ± 1.17 ng/mL [139]	subclinical 1.48 ± 0.93 ng/mL [139]	TSH [138]	Nd.
		control 0.95 ± 0.75 ng/mL [139]		TSH level is not correlated with vaspin level [139]	
**omentin**	↓ ~30 µg/L [127]	↓ (before thyroid hormone therapy) [141]		Nd.	fT_3_ [127]

**↑**—increased compared to euthyroid control, **↓**—decreased compared to euthyroid control, **THs**—thyroid hormones, **TSH**—thyroid-stimulating hormone, **T_3_**—triijodothyronine, **fT_3_**—free triiodothyronine, **T_4_**—thyroxine, **fT_4_**—free thyroxine, **TT_4_**—total thyroxine, **Nd.**—no data.

In conclusion, the results of this study suggest that the levels of metabolic hormones, including adipokines, change with the occurrence of thyroid dysfunction (hyperthyroidism and hypothyroidism). Furthermore, the correlations shown between blood levels of THs and metabolic hormones confirm that metabolic hormone levels are regulated by THs. This finding provides a basis for the hypothesis that THs may exert an indirect influence on the progression of OC, possibly through the regulation of metabolic hormones whose effects on OC progression have been described. However, further molecular studies demonstrating this effect are needed to confirm this hypothesis.

## 8. Correlation Between the Treatment of Thyroid Dysfunction and Ovarian Cancer

### 8.1. Effects of Thyroid Dysfunction Treatments on Ovarian Cancer Risk and Progression

The standard treatment for patients with thyroid dysfunction is antithyroid medication, radioactive iodine ablation, and synthetic iodine or surgery [30,142]. Studies by Minlikeeva et al. [39] reported that medications used for hyperthyroidism or hypothyroidism were not associated with mortality among OC patients. A study conducted by Leung et al. [42] revealed that there was no statistically significant risk of developing gynecological cancers, including OC, in patients with hyperthyroidism or hypothyroidism after medication adjustment. Nevertheless, the impact of interferon administration on patients diagnosed with hepatitis C [143], particularly women afflicted with thyroid disease, may be associated with the development of OC [72]. In addition, Muderris et al. [144] reported that patients with hypothyroidism had significantly larger ovarian volumes than controls did, which were significantly reduced after TH therapy.

### 8.2. Effects of Treatments for Ovarian Cancer on Thyroid Dysfunction

In a 50-year-old woman diagnosed with stage III HGSC, after surgery and complete therapy, the TSH level was undetectable, and the fT_4_ level was elevated, confirming the diagnosis of Graves’ disease [145]. A 55-year-old female with OC that metastasized to the thyroid resulting in clinical hypothyroidism was reported by Skarf et al. [146]. Kimura et al. [147] reported a 31-year-old female patient with massive ascites and elevated serum CA 125 levels and no typical features of hypothyroidism.

## 9. Ovarian Cancer, Including Thyroid Tissue (Struma Ovarii)

Thyroid tissue may be present in OC, known as struma ovarii (SO). GeCTs account for approximately 20% of all OC cases, with up to 20% of OC cases containing thyroid tissue. However, only 5% of these cancers contain more than 50% thyroid tissue and are termed SO [148]. Notably, the vast majority of SO cases (95%) are benign, with only a small percentage (5–20%) associated with hyperthyroidism [149]. In rare instances, malignant thyroid tumors can develop within the SO, which can lead to hyperthyroidism due to the capacity of the thyroid tissue within the tumor to produce THs. Hypothyroidism, on the other hand, occurs in 51.5% of SO patients and is more prevalent in benign SO patients than in malignant patients [150]. Thyroid carcinoma on struma ovarii (TCSO) is a rare OC derived from monodermic teratomas. It represents approximately 0.01% of all OCs and 5–10% of all SOs [151]. A case report by Koehler et al. [152] described a case of a 61-year-old female patient with Hashimoto’s thyroiditis on levothyroxine therapy who progressed to clinical and biochemical hyperthyroidism despite antithyroid medication, with a new diagnosis of a mature teratoma composed predominantly of thyroid tissue with high levels of sodium iodide symporter protein expression. Following the surgical resection of SO and the subsequent resolution of autonomous TH production, the patient presented symptoms consistent with hypothyroidism, as evidenced by a significant decrease in TSH levels of both fT_4_ and fT_3_. This finding suggests secondary hypothyroidism, a diagnosis that is substantiated by the known coexistence of chronic lymphocytic thyroiditis in the orthotopic thyroid gland [152]. Furthermore, hypothyroidism in conjunction with OC has been documented in patients after SO tumor resection [153].

## 10. Conclusions

In conclusion, in this review, we sought to elucidate the relationships between the presence of hyperthyroidism or hypothyroidism and OC. This is very important, especially in the context of the discovery of the expression of the nuclear TRs (TRα and TRβ), as well as the membrane TR (αvβ3 integrin), in human tissues and OC cell lines. The presented data from the database revealed that changes in TR expression levels regulate survival in patients with OC. The results of these studies demonstrated that TH, through binding to these receptors, may facilitate the progression, metabolism, and metastasis of OC, confirming the correlation between thyroid dysfunction and the occurrence of OC.

Given this connection, clinicians treating OC patients with known thyroid dysfunction should consider how treating this condition might affect the effectiveness of OC treatment. Furthermore, compelling evidence may encourage gynecologic oncologists to consider thyroid status when evaluating OC patients, potentially allowing more personalized clinical management of these patients. Interventional studies could facilitate the development of novel, effective treatments, which are currently lacking in OC therapy.

However, further molecular studies are needed to elucidate the mechanism by which the HPT axis promotes the development of OC to improve the efficacy of treatment and the quality of life of OC patients with hyper- or hypothyroidism.

## Figures and Tables

**Table 1:** The effects of hyperthyroidism and hypothyroidism on OC risk and mortality.

Thyroid Dysfunction	Incidence and Mortality	HR and Cl	Number of OC Patients	Authors
**HYPERTHYROIDISM**	↑ almost twice the risk of OC in women aged 20–69 years	HR:1,8; (1.0–3.0) 95% CI	767 and 1367 controls	[3]
	positive correlation with incidence of OC in women aged 29–50 years	Nd.	12	[37]
	↑ risk of OC incidence in women relative to controls even after 5 years of follow-up	HR: 1.31; (1.13–1.52) 95% CI	404 and 578 controls	[38]
	↑ risk of OC positively correlates with genetic predisposition to hyperthyroidism ↑ mortality in patients with OC	HR: 1.094; (1.029–1.164) 95% CI	1218 and 198,523 controls several	[4]
	↑ OC mortality in women treated with radioactive iodine compared to women with a healthy thyroid in the control group ↑ risk of death from OC	HR: 5.33; (2.17–13.08) 95% Cl HR: 1.65; (0.81–3.37) 95% Cl	5 and 143 controls several	[5]
	↑ risk ratio for OC mortality	HR: 2.14; 95% Cl;	1501 and 69,119 control	[40]
	↑ mortality associated with diagnosis of hyperthyroidism 5 years prior to OC diagnosis positive correlation with worse 5-year OC survival − association with overall OC mortality at 5 years of follow-up	HR: 1.94; (1.19 –3.18) 95% CI	160	[39]
	− risk of developing OC	HR: 0.67; (0.30–1.49) 95% CI	6 and 1052 controls	[41]
	↓ risk of developing gynecological cancers, including OC	Nd	44 852	[42]
**HYPOTHYROIDISM**	↑ slightly risk of developing OC	Nd	296 872	[42]
	medium association with OC mortalityworse 5-year OC survival	HR: 1.16; (1.03–1.32) 95% CI	several 624	[39]
	− risk of developing OC	HR: 0.84; (0.68–1.04) 95% CI	92 and 1052 controls	[41]
	− OC incidence	Nd	1218 and 198,523 controls	[4]
	− risk of death from OC	HR: 1.35;95% CI	4456 and 69,119 control	[40]

**OC**—Ovarian cancer, ↑—significant increase, ↓— significant decrease, **−**—no association, **HR**—hazard risk, **CI**—confidence interval, **Nd**—no data.

## Data Availability

No new data were generated for this review.
